# Histopathologic Responses of the Dental Pulp to Calcium-Enriched Mixture (CEM) and Mineral Trioxide Aggregate (MTA) in Diabetic and Non-Diabetic Rats

**Published:** 2014

**Authors:** Zahra Sadat Madani, Azam Haddadi, Abbas Mesgarani, Maryam Seyedmajidi, Amrollah Mostafazadeh, Ali Bijani, Manouchehr Ashraphpour

**Affiliations:** 1 *Dental Materials Research Center Dental School, Babol University of Medical Sciences, Babol, Iran.*; 2 *Dental School, Mazandaran University of Medical Sciences, Sari, Iran.*; 3 *Dental School, Babol University of Medical Sciences Babol, Iran.*; 4 *Cellular& Molecular Biology Research Center, Babol University of Medical Sciences, Babol, Iran.*; 5 *Non–Communicable Pediatric Diseases Research Center, Babol University of Medical Sciences, Babol, Iran.*; 6 *Department of pharmacology and Neurosciences, Babol University of Medical Sciences, Babol, Iran.*

**Keywords:** Diabetes, direct pulp capping, CEM cement, MTA

## Abstract

Diabetes mellitus is a chronic disease which affects the healing ability of the pulp and periodontium. The aim of the present study was to assess the histopathologic response of dental pulp to pulp capping using MTA or CEM cement in diabetic rats. Thirty two Wistar male rats aged between 8 and 10 weeks (weight: 200-250g) were divided into two groups of diabetic (n=16) and healthy (n=16) animals and then subdivided into MTA and CEM subgroups. In each group, 10 MTA treated, 10 CEM treated and 12 intact (without any intervention) teeth were analyzed. Intact teeth were considered as a baseline inflammation control. Then, class I cavity was made in the maxillary first molars teeth with pinpoint pulpal exposure. Either MTA or CEM cement was then placed over exposed pulp as pulp capping agent and the cavities were restored using resin- modified glass ionomer cement. Both teeth of rats in subgroups remained intact without any intervention. After four weeks, the rats were sacrificed and the teeth were subjected to histological evaluation in terms of inflammation intensity, dentin bridge formation and dentin bridge continuity. The CEM cement treated diabetic rats exhibited a significant higher inflammatory response when compared to healthy control group (P=0.004) whereas, MTA treated diabetic rats did not exhibit a significant higher inflammatory response in comparison to healthy controls. There was no significant difference between MTA and CEM cement in the induction of dentin bridge formation in diabetic and healthy controls. This preliminary study suggests that MTA is a superior dental material than CEM cement for pulp therapy in subjects with diabetes.

As a multisystem metabolic disease, dianetes tmellitus (DM) alters many organ and tissue functions including dental pulp and periapical tissues (1). Moreover, this disease can also affect the endodontic treatment outcome (2). There is accumulating evidence implicating that diabetes influences the healing process of periradicular lesions. A direct positive correlation has been found between serum glucose levels and the size of the periradicular lesions (2-4). Also, in a diabetic rats model, the periradicular lesions were greater in comparison to rats with normal glucose level despite the absence of infection in the diabetic group (5). Because of mutual effects of inflammation and hyperglycemia on each other, the control of infection and inflammation can be considered a necessary measurement in diabetic patient management. Indeed, the goal of endodontic treatment is to maintain the vitality of pulp in addition to debridement, cleaning, shaping and root canals obturation which subsequently result in the diminution of dental pulp inflammation. The success rate of this treatment depends on some factors such as; patient’s general health, dental pulp tissue condition and the type of pulp capping materials used (6, 7). Calcium hydroxide is generally used for direct pulp capping procedure. This substance can induce different levels of inflammation that could either result in amorphous dentin bridge formation or pulpal necrosis (based on the pH of calcium hydroxide) (8, 9). Mineral trioxide aggregate (MTA) is another alternative substance which is routinely used for pulp capping in clinical practice. MTA is composed of tri-calcium silicate particles, tri-calcium aluminum, tri-calcium oxide and silicate oxide (10). Several studies showed that in healthy subjects, an odontoblastic layer was formed following the use of MTA in pulpectomized teeth accompanied by no inflammation to mild inflammation and formation of uniformly thick dentin bridges with less porosity (11-15). Calcium enriched mixture (CEM) cement has recently been introduced in dentistry with a different composition compared to MTA (16). Shorter setting time, minimal discoloration and easy handling are among the desirable characteristics of CEM cement compared to MTA (16). Asgari et al. have shown that in comparison to MTA, when CEM cement is used for direct pulp capping, a better pulpal response is exhibited and a thicker dentin bridge is formed (17, 18). However, this finding was not confirmed in the study of Zarrabi and Tabarsi (14, 19). This study was designed to examine the effects of these two substances on dental pulp regeneration in diabetic and non- diabetic rats. The results generated by this study should provide a reliable basis to select a proper dental capping material for patients with diabetes mellitus.

## Materials and Methods

This study was approved by the Ethics Committee of Babol University of Medical Sciences, Babol, Iran (code: 5895). Thirty-two Wistar male rats aged between 8 to 10 weeks (weight: 200-250 g) were divided into two groups of healthy (n=16) and diabetic (n=16) animals and then subdivided into MTA and CEM subgroups. In each group, 10 MTA treated, 10 CEM treated and 12 intact (without any intervention) teeth were analyzed,.The intact teeth were considered as a baseline inflammation control.

To induce hyperglycemia in the diabetic group, 65 mg/kg Streptozotocin was injected intraperitoneally and blood glucose level was measured after one week. The rats with fast blood sugar FBS>200 mg/dL were considered as eligible cases for study according to Kohsaka et al. and Garber et al. (5, 20).

The rats were anesthetized via intraperitoneal injection of 80 mg/ kg of ketamin hydrochloride and 2.5 mg/kg xylazin 2%. The maxillary first molar in each rat was then rinsed with 0.12% chlorhexidine and class I cavity was prepared with pulpal exposure using a high speed handpiece (NSK, Japan) and a round diamond bur 1/16 (juta-swiss) Once the pulp was exposed, 5.25% sodium hypochlorite (Tage-Donyayearayesh, Iran) was used for hemostasis. MTA (AWMTA; Angelus, Londrina, PR, Brazil) and CEM Cement (Bionique dent, Tehran, Iran) were then prepared according to the manufacturer’s instruction and placed over the exposed areas in the cavities. The cavities were then restored using glass ionomer cement (GC International Corp, Tokyo, Japan) and the rats were sacrificed after four weeks.

The jaws were separated and fixed in 10% formalin solution for 1 week. Then they were inserted in formic acid 10% for decalcification. After that, the teeth were cut vertically in equal halves and paraffined blocks were prepared from specimens. 6 µm thick slices were cut from blocks and the sections were stained using hematoxylin and eosin (H&E). Histopathological evaluation was performed in three fields under Olumpus BX41 light microscope (Olympous, Tokyo, Japan) at 40X magnification. The evaluation was performed by a pathologist who was blind to the experimental groups. The sections were evaluated in terms of the type of inflammation, the number of inflammatory cells (polymorphonuclear/ mononuclear cells), and dentin bridge formation based on Campe et al. reported criteria (Table 1) (21).

The data were analyzed by SPSS version 17. T-test was used to compare the quantity of inflammatory cells in the healthy and diabetic groups and one way ANOVA was used to compare the number of inflammatory cells in MTA, CEM cement and the control groups and dentin bridge formation and its location among the healthy and diabetic groups was compared using Chi-Square test.

## Results

Thirty-two rats (16 healthy and 16 diabetic rats) were studied. All rats showed some degree of chronic inflammation. The results revealed significant difference in the intensity of inflammation between the healthy and diabetic groups (P= 0.037). There was no significant difference in the continuity of the dentin bridge among the healthy and diabetic groups (P= 0.585 and P= 0.313, respectively).

The mean number of inflammatory cells in the diabetic group and the healthy group was 38.67±5.70 and 17.83±5.34, respectively. This difference was statistically significant (P= 0.01). One way ANOVA test failed to reveal any significant difference among the number of inflammatory cells in two treatment groups (MTA, CEM cement) in the healthy (P= 0.571) and diabetic (P= 0.068) groups.

**Table 1:** Criteria used for histopathological evaluation

**Type**	**0 (no inflammation)**	**1 (acute inflammation)**	**2 (chronic Inflammation)**	**3 (chronic and acute inflammation)**
Intensity	No inflammation	Mild(inflammatory cells<30)	Intermediate (30<Inflammatory cells<60)	Intense (inflammatory cells>60)
Position	Direct contact with the capping material	Close proximity with the material	No evidence of dentin bridge formation	-
Continuity	Complete	Incomplete	Absence of dentin bridge	-

**Inflammatory/anti inflammatory effects of MTA and CEM cement in pulp treatment**

To investigate the possible inflammatory/ anti-inflammatory effect of MTA and CEM cement, the basal levels of inflammation of rat pulp tissues were determined in 12 teeth prepared from each diabetic and healthy group and then compared to post MTA and CEM treatment inflammation levels in these animals.

**Fig. 1:**
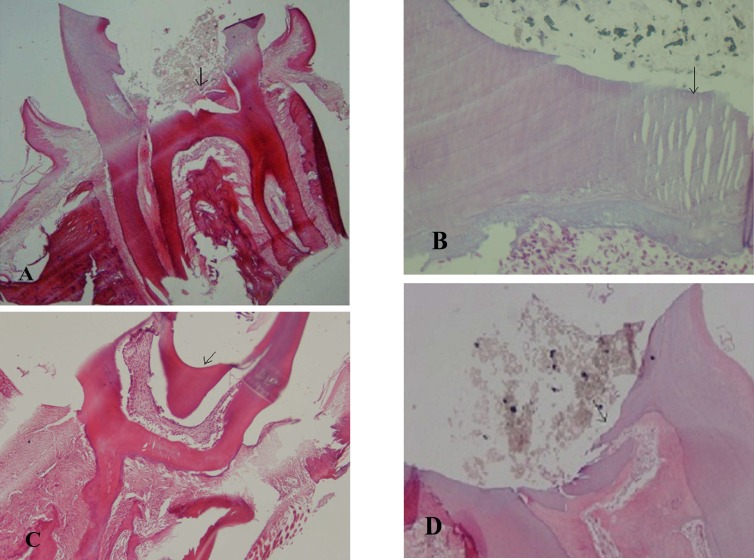
Histological findings at 4 weeks: Photomicrographs showing the presence of inflammation by H&E staining. A,B: mild inflammation in the diabetic (x400) and healthy groups (x40) respectively treated with MTA. C, D: intense © and mild (D) inflammation in the diabetic and healthy groups respectively treated with CEM (x40). Arrows indicate inflammatory cell

Interestingly, we observed that 4 teeth from each group exhibited intense inflammation and have been discarded in all healthy rats’ teeth but not in diabetic rats’ teeth after treatment with both MTA and CEM cement (Table 2). As it can be see in Figure.1 (A-D), the overall basal levels of inflammation in diabetic rats’ teeth were higher in comparison to healthy rats’ teeth. However, this difference was not statistically significant in two treatment groups (MTA, CEM cement). After dental pulp treatment with MTA and CEM cement, this basal inflammation diminished in both groups, but this reduction was higher in healthy rats. Almost all healthy rats exhibited a mild dental pulp inflammation (Table 2).

**Table 2:** Teeth characteristics in different groups

**Group**	**Variable**	**Grade**	**Healthy** *	**Diabetic** *
MTA treated teeth	Intensity of inflammation	Mild	9	6
		Intermediate	1	2
		Intense	0	2
		p=0.063	n=10	n=10
	Continuity of the dentin bridge	Absence of dentin bridge	0	2
		Intermittent	6	5
		Continuous	4	3
		p=0.500	n=10	n=10
	Position of the dentin bridge	In contact with the material	10	8
		No dentin bridge formation	0	2
		p=0.474	n=10	n=10
CEM cement Treated teeth	Intensity of inflammation	Mild	9	4
		Intermediate	1	3
		Intense	0	3
		P= 0.004**	n=10	n=10
	Continuity of the dentin bridge	Absence of dentin bridge	0	2
		Intermittent	7	6
		Continuous	3	2
		P= 0.628	n=10	n=10
	Position of the dentin bridge	In contact with the material	10	8
		No dentin bridge formation	0	2
		P= 0.474	n=10	n=10
Intact teeth	Intensity of inflammation	mild	8	5
		Intermediate	0	3
		Intense	4	4
		P= 0.347	n=12	n=12

* indicate the number of tooth

** P< 0.05 was considered as a statistically significant level

Both MTA and CMA cement exhibited anti-inflammatory effects in rat pulp treatment. However, MTA appeared to be more effective in this context especially in the treatment of diabetic rats’ dental pulp.

Both MTA and CEM cement exhibited anti-inflammatory effects in rat pulp treatment. However, MTA appeared to be more effective in this context especially in the treatment of diabetic rats’ dental pulp. As it can be deduced from table 2, the median value of inflammation intensity in MTA treated diabetic rats’ teeth was the same as in MTA treated healthy rats; i.e. more than 50% of animals in both groups exhibited a mild pattern of inflammation. This value in CEM cement treated healthy rats’ teeth was similar to that of MTA treated groups. However, more than 50% of CEM cement treated diabetic rats exhibited an intermediate pattern of inflammation. Moreover, the intensity of inflammation in CEM cement treated diabetic rats’ teeth was significantly higher than the CEM cement treated healthy rats (P=0.004) whereas, we were not able to see any significant difference for this value between the diabetic and healthy rats when they were treated with MTA (Table 2).

**Effect of MTA and CEM cement on rat dentin regeneration**

**Fig. 2:**
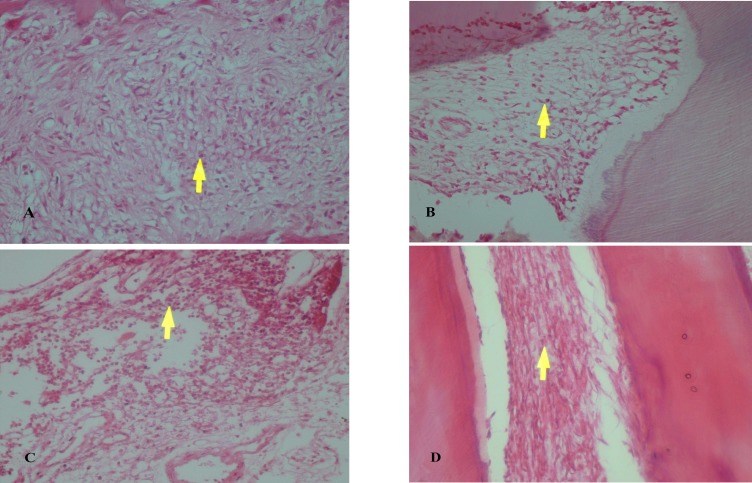
Histological findings at 4 weeks: Photomicrographs showing dentin bridge formation by H&E staining (x40). A,B: MTA treatment in diabetic and healthy groups respectively. C, D: CEM treatment in diabetic and healthy groups respectively. Arrows indicate dentin bridge

To compare the capacity of MTA and CEM cement in hard tissue repair, we evaluated dentin bridge formation after MTA and CEM treatment in diabetic and healthy rats. In diabetic rats treated with either MTA or CEM cement, two teeth showed no dentin bridge formation. Seven teeth in the MTA group and 5 teeth in the CEM group showed evidence of complete dentin bridge formation. However, the statistical analysis comparing the healthy and diabetic rats failed to show any significant difference in dentin bridge formation among the groups treated with MTA or CEM cement (P> 0.05) (Table 2). Figure 2 shows the dentin bridge formation in MTA and CEM treated diabetic and healthy rats’ teeth.

## Discussion

It is likely that the number of diabetic patients requiring endodontic treatment will increase (22). In one hand, the chronic elevated level of glucose which exists in patients with diabetes makes them susceptible to the systemic inflammation through damaging mitochondrion and mitochondrial DNA (23), in another hand, the elevated levels of inflammation can increase the blood glucose levels and consequently intensify the diabetes (24, 25(. Moreover, these patients exhibit some degree of defect in tissue regeneration and wound healing process (22). Hence, the removing of the infectious germs and inflamed tissues by pulp treatment and then exerting the pulp capping procedure by a proper biomaterial with the property of hard tissue regeneration induction could be considered as a beneficial measure for these patients. MTA and CEM cement are two dental materials which have a variety of applications in endodontics including direct pulp capping. CEM cement is a material with many desired characteristics such as biocompatibility, induction of mineralization and setting in the presence of moisture (26-30). In addition to sharing the advantages of MTA, the other benefits of CEM cement include ease of handling, cost effectiveness and minimal tooth discoloration (16, 31-33). This study was designed to examine the inflammatory effects as well as the dentin regeneration induction capacity of these substances in a rat model of diabetes. The data generated by this study showed that CEM cement induced higher levels of inflammation in diabetic rats when compared to the inflammation levels in healthy animals. We were not able to find any significant difference in the levels of MTA induced inflammation between diabetic and healthy animals. We also found that after treatment with both materials, the basal level of inflammation did not only increase, but also in MTA, and the CEM cement treated healthy rats as well as the MTA treated diabetic rats it was even reduced to mild level and in CEM cement treated diabetic rats it was decreased to intermediate level (Table 2). Although all of these differences were not statistically significant. We suppose that the short follow-up time of the present study (one month) is not enough for this difference to appear at a statistically significant level. We were also not able to find any significant difference in basal inflammation levels in intact teeth controls between the diabetic and healthy rat groups. It is well known that there is a higher inflammation level in diabetic condition (24**).** However**,** the main question which should be answered here is why the diabetic rats exhibited a statistically significant higher level of CEM cement post-treatment inflammation response than healthy animals. We assumed that under hyperglycemic condition, MTA may protect the pulp tissue with more efficacy than CEM cement against injury induced inflammation which occurrs when the dentin is cut in order to expose the pulp. To our knowledge, this is the first study which compares the CEM cement and MTA capacity in inflammation induction in diabetic and healthy conditions in rat model.

After tooth pulp injury, progenitor cells migrate to the pulp tissues and differentiate into second generation of odontoblasts (34). These cells produce new dentin called secondary dentin. In this study, we investigated the dentin bridge formation as well as its position as markers to evaluate the MTA or CEM cement potential in dentin regeneration induction in diabetic and healthy rats. Moreover, by the Pearson statistical test, the correlation between dental bridge formation and its position with the level of dental pulp inflammation was determined. We found that the two substances were equally able to induce the dentin regeneration in both diabetic and healthy rats. We were not able to find a significant correlation between dentin bridge formation and the pulp inflammation levels. To the best of our knowledge, this the first study which compares the CEM cement and MTA capacity in dentin bridge induction in diabetic and healthy conditions in rat model.

Taken together, the data generated from this study showed that in rat model, both MTA and CEM cement have the potential to induce dentin regeneration at a proper level. However, MTA had a significantly lower capacity to induce inflammation in diabetic rats.

From this preliminary study, it could be concluded that MTA is a superior dental material than CEM cement for pulp therapy in diabetic subjects.
